# Supplementary material for: The Value of Micro-CT in the Diagnosis of Lung Carcinoma: A Radio-Histopathological Perspective
Source: Diagnostics (Basel). 2023 Oct 20;13(20):3262. doi: 10.3390/diagnostics13203262 (PMC10606474; doi:10.3390/diagnostics13203262)
Supplement: Supplementary file 1 [file diagnostics-13-03262-s001.zip › Supplementary video descriptions.pdf]

Supplement video descriptions

Supplement video 1: Micro-CT scanning of FFPE block of a pulmonary adenocarcinoma

Supplement video 2: Non-tumoral fresh lung tissue obtained from a lobectomy specimen performed for an adenocarcinoma lobectomy.

The specimens were scanned with a high-resolution desktop micro-CT system (Bruker micro-CT Systems 1275, Kontich, Belgium). The scanning parameters used were: 35 kVp, 231-mA, without filter, 16 µm pixel size, rotation at 0.2 steps with 360°. The mean time of scanning was around 30 minutes.

NRecon (ver. 1.7.4, Bruker micro-CT Systems, Kontich, Belgium) software was used for the reconstruction. CTAn (v. 1.20.3 Bruker micro-CT Systems, Kontich, Belgium) software was used for the 3D image analysis. CTVox (v. 3.3.0 Bruker micro-CT Systems, Kontich, Belgium) was used for visualization of the samples for movies.
